# Pre-silencing of genes involved in the electron transport chain (ETC) pathway is associated with responsiveness to abatacept in rheumatoid arthritis

**DOI:** 10.1186/s13075-017-1319-8

**Published:** 2017-05-25

**Authors:** C. Derambure, G. Dzangue-Tchoupou, C. Berard, N. Vergne, M. Hiron, M. A. D’Agostino, P. Musette, O. Vittecoq, T. Lequerré

**Affiliations:** 1 Normandie Univ, UNIROUEN, Inserm U 1245, F 76000 Rouen, France; 2 Normandie Univ, UNIROUEN, Inserm U 905, F 76000 Rouen, France; 3 LITIS EA 4108, Computer science, information processing and systems laboratory, Normandy University, Institute for Research and Innovation in Biomedicine, 76451 Mont-Saint-Aignan, France; 4 LMRS UMR 6085 CNRS, Raphaël Salem laboratory, Normandy University, 76575 Saint Étienne du Rouvray, France; 5 Departement of Rheumatology, AP-HP Ambroise Paré Hospital, University of Versailles Saint Quentin en Yvelines, 92100 Boulogne-Billancourt, France; 6 Normandie Univ, UNIROUEN, Inserm U 1234, Rouen University Hospital, Department of Dermatology, F 76000 Rouen, France; 7 Normandie Univ, UNIROUEN, Inserm U 1234, Inserm CIC-CRB 1404, Rouen University Hospital, Department of Dermatology, F 76000 Rouen, France

**Keywords:** Biomarkers, Rheumatoid arthritis, Abatacept, Microarray, Abatacept response, Oxydative stress, Mitochondria

## Abstract

**Background:**

In the current context of personalized medicine, one of the major challenges in the management of rheumatoid arthritis (RA) is to identify biomarkers that predict drug responsiveness. From the European APPRAISE trial, our main objective was to identify a gene expression profile associated with responsiveness to abatacept (ABA) + methotrexate (MTX) and to understand the involvement of this signature in the pathophysiology of RA.

**Methods:**

Whole human genome microarrays (4 × 44 K) were performed from a first subset of 36 patients with RA. Data validation by quantitative reverse-transcription (qRT)-PCR was performed from a second independent subset of 32 patients with RA. Gene Ontology and WikiPathways database allowed us to highlight the specific biological mechanisms involved in predicting response to ABA/MTX.

**Results:**

From the first subset of 36 patients with RA, a combination including 87 transcripts allowed almost perfect separation between responders and non-responders to ABA/MTX. Next, the second subset of patients 32 with RA allowed validation by qRT-PCR of a minimal signature with only four genes. This latter signature categorized 81% of patients with RA with 75% sensitivity, 85% specificity and 85% negative predictive value. This combination showed a significant enrichment of genes involved in electron transport chain (ETC) pathways. Seven transcripts from ETC pathways (*NDUFA6*, *NDUFA4*, *UQCRQ*, *ATP5J*, *COX7A2*, *COX7B*, *COX6A1*) were significantly downregulated in responders versus non-responders to ABA/MTX. Moreover, dysregulation of these genes was independent of inflammation and was specific to ABA response.

**Conclusion:**

Pre-silencing of ETC genes is associated with future response to ABA/MTX and might be a crucial key to susceptibility to ABA response.

**Electronic supplementary material:**

The online version of this article (doi:10.1186/s13075-017-1319-8) contains supplementary material, which is available to authorized users.

## Background

Recent advances in our understanding of the pathophysiology of rheumatoid arthritis (RA) have led to the development of new biologic treatments designed to act against a precise therapeutic target: these include tumor necrosis factor-alpha (TNFα), interleukin-1-receptor antagonist (IL1-Ra, anakinra), cytotoxic T-lymphocyte-associated protein 4 (CTLA4-Ig or abatacept (ABA)), CD20 expressed on B cells (rituximab) and IL-6 receptor (tocilizumab) [1]. All these biologic agents have proven efficacy in stopping joint inflammation and structural damage in association with the anchor drug methotrexate (MTX) [1]. No one molecule has proven its clinical superiority over others in terms of efficacy [2–6]. In addition, no response to these treatments is obtained in approximately 30% of patients with RA, and the response to all these medications is highly variable from one patient to another. Nevertheless, prescription of biologics agents remains highly empirical [7–9]. Presently, we are still unable to predict the clinical efficacy of these treatments in a given patient because of the heterogeneity of RA and the subgroups of patients susceptible to responding better to one molecule than to another. Given the increasing number of available molecules, it is crucial to identify predictive markers in order to optimize drug prescription only to those patients susceptible to responding and thus avoiding side effects.

In the current context of personalized medicine, large-scale analysis of gene expression to predict drug response is a relevant and original approach, which has already shown its utility in cancer or kidney transplantation [10–12]. Gene expression profiling is clearly a powerful method for the identification of biomarkers and the development of personalized medicine in the field of rheumatology [13–16]. This approach has already allowed us to identify and validate two gene combinations able to predict response to infliximab or anakinra although in small cohorts of patients with RA [17, 18]. The proof of concept of gene expression profiles or signatures as a predictor of good response to drugs in RA was further confirmed by other teams using other biologic agents, but in cohorts that included very few patients with RA [19–21]. Thus, we conducted this present ancillary study as a follow-up to the APPRAISE trial [22, 23]. Our main objective was to identify and validate a gene expression profile associated with a good response to ABA/MTX, and to understand the involvement of this signature in RA pathophysiology.

## Methods

### Patients from the APPRAISE trial

A total of 68 patients with RA from the APPRAISE trial were enrolled in this ancillary study [22, 23]. The APPRAISE study assessed the capability of the composite power of Doppler and greyscale ultrasound score to measure the early effect and time course of response to treatment with ABA in biologic-naïve patients with active RA despite MTX therapy. APPRAISE (NCT00767325) initially including 104 patients with RA was a 24-week, Phase IIIb, open-label, multicenter, single-arm study conducted at 21 sites across Europe (Denmark, France, Germany, Hungary, Italy, Norway, Spain and the UK) [22, 23]. Eligible patients were ≥18 years of age, had American College of Rheumatology (ACR)-defined RA according to the 1987 classification criteria for at least 6 months [24], and had been on MTX (≥15 mg/week) for at least 3 months prior to baseline, with a stable MTX dose for at least 28 days before baseline (except in cases of intolerance to MTX). Patients were required to have active disease, defined by a baseline disease activity score in 28 joints (calculated with C-reactive protein (CRP)) (DAS28(CRP)) >3.2 or tender and ≥6 swollen joint counts, and CRP above the upper limit of normal. All patients received intravenous (IV) infusions of ABA at a weight-titered dose of 10 mg/kg at baseline (day 1), and at weeks 2, 4, 8, 12, 16, 20 and 24, in addition to stable doses of concomitant MTX (≥15 mg/week). MTX dose increases were not permitted, and dose decreases were allowed only in cases of intolerance. Oral corticosteroid use (stable dose of ≤10 mg prednisone/day) was permitted during the study. For this study, 5 ml of whole blood was collected in PAXgene RNA tube (PreAnalytiX, Qiagen) just before the first infusion and 6 months later and was stored at –80 °C until use.

### Clinical evaluation and response to ABA/MTX

Several clinical characteristics were collected at baseline and 6 months later: age, gender, disease duration and MTX and corticosteroid doses. Disease activity was evaluated at all assessment visits (baseline, weeks 1, 2, 4, 6, 8, 12, 16, 20 and 24) using the DAS28(CRP) calculated from 28 tender joints, 28 swollen joints, CRP and patient global assessment (visual analog scale (VAS); 0–10 scale).

The response to ABA/MTX was evaluated at 6 months using European League Against Rheumatism (EULAR) response criteria based on DAS28(CRP) [25]. Since we were looking for biomarkers associated with good response to ABA/MTX, patients were categorized according to their EULAR response as responders (R) (*n* = 36) or non-responders (NR) (comprising moderate responders (*n* = 25) and no responders (*n* = 7)) [25].

### RNA preparation

Total RNAs from whole blood were extracted with PAXgene blood RNA kit according to the manufacturer’s recommendations (Qiagen PreAnalytiX GmbH, Courtaboeuf, France) and stored at –80 °C until use. Total RNA from 10 healthy donors (5 women and 5 men) was pooled and used as an internal standard reference (control pool). The quality and quantity of isolated mRNAs were assessed using the 2100 Bioanalyzer (Agilent Technologies, Santa Clara, CA, USA) and the Nanodrop device (Thermo Scientific, Wilmington, USA). Only RNA samples with a minimal RNA integrity number of 7 were used for subsequent experiments.

### Microarrays

Whole human genomic DNA microarrays were used to analyze two-colored gene expression profiling (4 × 44 K Whole Human Genome, Agilent Technologies, Les Ulis, France). Each RNA sample from patients with RA was labeled by Cyanine-5 and co-hybridized with a Cyanine-3 labeled RNA control pool according to the manufacturer’s instructions (Low Input QuickAmp Labeling Kit, Agilent Technologies, Les Ulis, France). Briefly, 100 ng of RNAs were labeled with cyanine-5 CTP (patients with RA) or cyanine-3 CTP (control pool). After hybridization reaction using a hybridization kit (Agilent Technologies, Les Ulis, France) co-hybridization was performed at 65 °C for 17 hours. After wash steps, the microarrays were scanned with a 5-μM pixel size using the DNA Microarray Scanner GB (Agilent Technologies, Les Ulis, France). Image analysis and extraction of raw and normalized signal intensities (lowess) were performed using Feature Extraction Software 10.5.1.1 (Agilent Technologies). The data were in agreement with the guidelines for minimum information about a microarray experiment and were deposited in the database of the National Center for Biotechnology Information Gene Expression Omnibus (https://www.ncbi.nlm.nih.gov/geo/query/acc.cgi?acc=GSE68215). The data are accessible [GEO:GSE68215]. Non-uniform spots and saturated spots or spots with intensities below the background were not taken into account. Only spots that passed these quality controls on 100% of arrays were selected for further analysis. Hierarchical clustering was performed using the Pearson coefficient metric and complete linkage to build the transcripts and sample dendrograms.

### Quantitative reverse transcription-PCR (qRT-PCR)

cDNA was synthesized from 1-μg RNA samples using random primers and M-MLV enzyme (Invitrogen™, Carlsbad, USA). qRT-PCR was performed using a Lightcycler as instructed by the manufacturer (Roche™, Meylan, France). qRT-PCR reactions were performed for each sample in duplicate using SYBR-Green (Roche™, Meylan, France) and values were normalized using the geometric mean of three control genes (*18S, ACTB, B2M*) defined by the geNorm algorithm [26]. Sequences of primers (Eurogentec™, Fremont, USA) used for qRT-PCR were: *BLOC1S1* forward, 5’-AAGCAGACAGGCCAGTGGAT-3’; *BLOC1S1* reverse, 5’-CAGTGCAGTGGCAATGGTG-3’; *RNASE3* forward, 5’-CAGGAGCCACAGCTCAGAGA-3’; *RNASE3* reverse, 5’-GAGCCCTCCACACCCATAAG-3’; *COX6A1* forward, 5’-CCACTTCCAACTGGCTACGA-3’; *COX6A1* reverse, 5’-AAGCAAAGGGATGGGAGACC-3’; *PTRH2* forward, 5’-GCTGTTGGAGTTGCTTGTGG-3’; *PTRH2* reverse, 5’-AGGCTGAAACAGCAGCATGA-3’; *18S* forward, 5’-GTGGAGCGATTTGTCTGGTT-3’; *18S* reverse, 5’-CGCTGAGCCAGTCAGTGTAG-3’; *ACTB* forward, 5’-CTGGAACGGTGAAGGTGACA-3’; *ACTB* reverse, 5’-AAGGGACTTCCTGTAACAATGCA-3’; *B2M* forward, 5’-TGCTGTCTCCATGTTTGATGTATCT-3’; *B2M* reverse, 5’-TCTCTGCTCCCCACCTCTAAGT-3’.

### Statistical and functional analysis

Comparisons of clinical and biological data between R and NR were performed at baseline using Student’s *t* test for continuous variables. Comparisons of R or NR before and after treatment were performed using the paired *t* test. Identification of clinical parameters able to predict good response to ABA/MTX was performed in two different multivariate analyses: (1) a logistic regression model with variable selection using Bayesian information criterion (BIC) and (2) linear discriminant analysis (LDA), which estimates a coefficient for each variable.

Data from transcriptomic analysis were analyzed using GeneSpring GX V.13.0 (Agilent Technologies, Les Ulis, France). The normality of log 2 ratio of gene expression was evaluated using the Shapiro–Wilk statistical test. The unpaired Student’s *t* test (*p* value < 0.05), with the Benjamini–Hochberg correction to check the false discovery rate (FDR), was used to determine the statistical significance of differences in gene expression levels between R and NR. Gene Ontology (GO) analysis was used to investigate the biological processes, molecular function or cellular localization enriched in the transcripts list, showing a significant fluctuation in gene expression between R and NR. The *p* value was computed by standard hypergeometric distribution. The GeneSpring Single Experiment Analysis (SEA) bio-informatics tool was used for computational analysis to identify potential curated canonical pathways with setting parameters (reactome and GenMAPP for pathway source), which are enriched in the differentially expressed transcripts list, using the WikiPathways database (http://www.wikipathways.org/index.php/Pathway:WP111). The significance of the association between the genes and the pathways was measured by Fisher’s exact test.

## Results

### Characteristics of patients with RA and their response to ABA/MTX

**Fig. 1:**
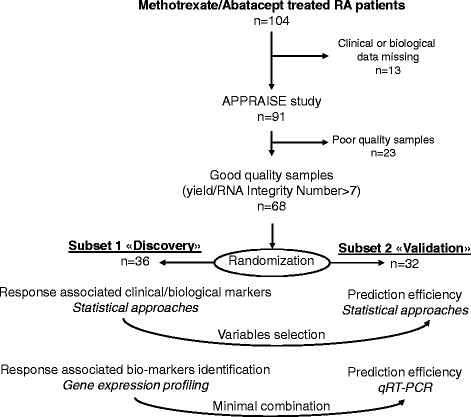
Ancillary study design from the APPRAISE trial. Of the 104 patients with rheumatoid arthritis (*RA*) enrolled in the APPRAISE trial, 68 were included in our ancillary study after discarding patients with missing data or poor-quality RNA samples. Among these 68 patients with RA, two subsets were designated, one for identification and one for validation. The first step of this ancillary study was to identify clinical parameters to predict abatacept (ABA)/methotrexate (MTX) response. The second step was to identify a gene combination able to predict ABA/MTX response. Subset 1, comprising 36 patients with RA, was used to identify clinical parameters or gene combinations to predict drug response. Subset 2, comprising 32 patients with RA, was used to validate these clinical parameters or gene combinations. *qRT-PCR* quantitative reverse-transcription PCR

Of the 104 patients with RA included in the original APPRAISE trial, clinical and biological data for subsequent analysis were available for 91 patients: 68 of these patients were recruited to this present ancillary study based on the quality of the RNA samples (Fig. 1). These 68 patients with RA were split into two subsets at random: subset 1 (n = 36) to identify clinical or biological (including transcripts) markers associated with response to ABA/MTX and subset 2 (n = 32) to validate a gene expression profile able to predict good response to ABA/MTX (Fig. 1). After 6 months of treatment, patients with RA were categorized according to their EULAR response as either responders (R: n = 17 and 19, respectively in subsets 1 and 2) or non-responders (NR: n = 19 and 13, respectively in subsets 1 and 2). Table 1 provides demographic and clinical information for these 68 patients with RA at baseline and after 6 months of treatment.

**Table 1:** Clinical and biological parameters of patients with rheumatoid arthritis

	Subset 1 (*n* = 36)				Subset 2 (*n* = 32)			
	R (*n* = 17)		NR (*n* = 19)		R (*n* = 19)		NR (*n* = 13)	
	0 months	6 months	0 months	6 months	0 months	6 months	0 months	6 months
Age (years)	49.94 +/- 3.63		57.68 +/- 2.67		55.32 +/- 3.02		58.46 +/- 3.32	
Sex (F/M), number	17/0*		14/5		15/4		10/3	
Disease duration (years)	3.71 +/- 0.86**		10.68 +/- 2.65		5.42 +/- 1.44**		12.85 +/- 3.1	
Methotrexate dose (mg/week)	15.29 +/- 1.28		16.58 +/- 3.99		18.03 +/- 3.85		15.58 +/- 3.42	
Corticosteroid dose (mg/day)	2.44 +/- 0.77		2.71 +/- 0.75		2.64 +/- 0.8		3.46 +/- 1.14	
Tender joint count/28	10.29 +/- 1.48	1.47 +/- 0.5^$$$^	14.42 +/- 1.46^£££^	6.37 +/- 0.79	11.10 +/- 1.56	1.32 +/- 0.37^$$$^	11.46 +/- 2.05	6.92 +/- 1.68^£££^
Swollen joint count/28	10.24 +/- 1.31	2.59 +/- 0.74^$$$^	11.26 +/- 0.92^£££^	6.21 +/- 1.10	9.63 +/- 1.15	1.74 +/- 0.49^$$$^	10.85 +/- 1.41	5.08 +/- 0.81^£££^
Patient assessment of disease (VAS scale/100 mm)	58.24 +/- 5.91*	16.29 +/- 4.37^$$$^	68.58 +/- 4.28^£££^	35.68 +/- 4.85	48.95 +/- 4.83*	19.74 +/- 3.87^$$$^	60.62 +/- 5.37	37.08 +/- 5.35^£££^
DAS28(CRP)	4.98 +/- 0.24**	2.37 +/- 0.13^$$$^	5.90 +/- 0.2^£££^	4.20 +/- 0.19	4.86 +/- 0.23**	2.41 +/- 0.11^$$$^	5.35 +/- 0.25	4.13 +/- 0.27^£££^
CRP (mg/L)	7.24 +/- 1.42**	2.18 +/- 0.44^$$$^	27.69 +/- 6.71^££^	12.34 +/- 3.46	8.16 +/- 2.44**	3.56 +/- 0.83^$$$^	16.64 +/- 5.39	11.38 +/- 2.46^££^

Clinical and biological variables were collected before treatment and 6 months later. Values are expressed as mean ± standard error of the mean unless stated otherwise. *R* responders, *NR* non-responders, *F* female, *M* male, *VAS* visual analog scale, *DAS* Disease Activity Score in 28 joints, *CRP* C-reactive protein. Response to abatacept/methotrexate was assessed by the DAS28 calculated with CRP at 6 months of treatment. Patients were categorized as indicated in “Methods”. *P* values were determined by Student’s independent samples *t* test or paired *t* test as appropriate: *p < 0.05; **p < 0.01 for comparisons between responders and non-responders at baseline; ^$$$^ *p* < 0.001 for comparison of values at baseline and 6 months in responders; ^££^ *p* < 0.01; ^£££^ *p* < 0.001 for comparison of values at baseline and 6 months in non-responders. All other comparisons were not significant.

The baseline characteristics of patients with RA from subsets 1 and 2 were comparable for all variables, suggesting absence of bias in randomization (Table 1). Whatever the subset, tender joint count (TJC), swollen joint count, global assessment of disease measured by the patient with the VAS, CRP and DAS28(CRP) improved significantly after 6 months of treatment in both R and in NR. However, the range of improvement was significantly higher in R than in NR for each parameter except the global VAS and CRP (Table 1 and Additional file 1). In each subset, there were statistical differences between R and NR at baseline in clinical and biological variables. Indeed, disease duration was longer in NR than in R (*p* < 0.01) (Table 1). DAS28, patient assessment of disease and CRP were higher in NR than in R (*p* < 0.05). Moreover, the sex ratio was significantly different between R and NR (*p* = 0.047). There was better response in patients with RA of short disease duration and moderate disease activity. These observations raise the question of using clinical and biological variables as predictors of response.

### Clinical and biological variables are not associated with response to ABA/MTX

**Fig. 2:**
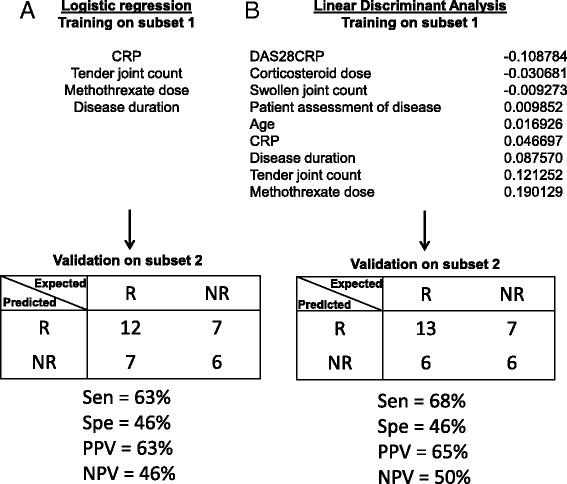
Inability of clinical and biological parameters to predict response to abatacept/methotrexate (ABA/MTX). Two independent statistical methods were performed to identify variables to predict drug response in subset 1. Next, the prediction accuracy of these variables was evaluated in subset 2. **a** In subset 1, four variables were selected by logistic regression: C-reactive protein (*CRP*), tender joint count, MTX dose and disease duration. In subset 2, these four variables allowed good categorization of 12 out of 19 responders (*R*) and 6 out of 13 non-responders (*NR*). The remaining 14 patients were misclassified. **b** In subset 1, linear discriminant analysis was performed to balance each parameter by calculation of the coefficient of linear discriminant analysis. In subset 2, these data allowed good categorization of 13 out of 19 R and 6 out of 13 NR. The remaining 13 patients were misclassified. *DAS28* disease activity score in 28 joints, *Sen* sensitivity, *Spe* specificity, *PPV* positive predictive value, *NPV* negative predictive value

Clinical and/or biological variables as predictors of response were identified by two different multivariate statistical approaches, logistic regression and linear discriminant analysis. Logistic regression based on more pertinent variables as MTX, TJC, disease duration and CRP (identified in subset 1) did not allow an accurate prediction of response to ABA/MTX in subset 2 (sensitivity = 63%; specificity = 46%) (Fig. 2a). Similarly, linear discriminant analysis, which balanced biological and clinical parameters, did not allow accurate prediction of response to ABA/MTX in subset 2 (sensitivity = 68%; specificity = 46%) (Fig. 2b). As a result of these shortcomings, we used a microarray approach to identify biomarkers associated with response to ABA/MTX.

### Gene expression profiling associated with response to ABA/MTX

**Fig. 3:**
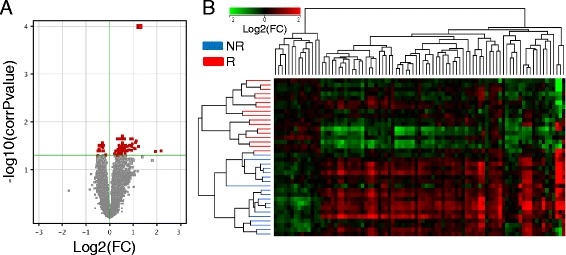
Gene expression profiling associated with response to abatacept/methotrexate in subset 1. Baseline samples from subset 1 (*n* = 36 (17 responders (*R*) and 19 non-responders (*NR*)) were hybridized on 4 × 44 K whole human genome microarrays. **a** The *t* test with the Benjamini–Hochberg correction for estimation of the false discovery rate (*p* < 0.05) was used to identify 87 transcripts (*red dots*) with dysregulation that was associated with response (R vs NR). **b** Hierarchical clustering: Pearson coefficient and complete linkage metrics were used to obtain the transcripts and sample dendrograms. Fold change (*FC*) is the ratio of relative abundance of transcripts in patients with rheumatoid arthritis vs control pool

With subset 1, the cRNA from 17 R and 19 NR were co-hybridized with a cRNA internal reference from 10 healthy subjects on whole human genome microarrays, by two-color technology. After elimination of spikes and flagged probes, 19,620 probes were detected in all samples. From these 19,620 transcripts, we identified 87 transcripts with statistically significant variation between R and NR according to the *t* test with correction for multiple testing (FDR, Benjamini–Hochberg correction) (Fig. 3a). These transcripts are listed in Additional file 2 (69 transcripts are referenced with a Ref Seq accession number while 18 probes were unknown). Since the sex ratio in R and in NR was significantly different, this maldistribution between male and female patients did not introduce bias into our study (data not shown) Finally, we performed hierarchical clustering of the 36 patients from subset 1 based on the levels of the 87 transcripts indicated previously, resulting in almost perfect separation of R and NR into two major clusters. Indeed, only one R patient was misclassified in the patient’s dendrogram (Fig. 3b).

**Fig. 4:**
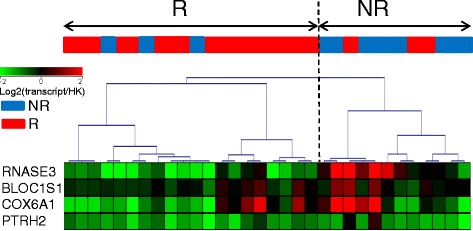
Validation of a small transcript combination associated with response to abatacept/methotrexate (ABA/MTX) in subset 2. A minimal combination of four transcripts (*RNASE3, BLOC1S1, COX6A1, PTRH2*) was identified by logistic regression from the 87 transcripts previously identified with subset 1. Next, the ability of these four transcripts to predict ABA/MTX response was evaluated by quantitative reverse-transcription (qRT)-PCR with subset-2 samples. Hierarchical clustering based on normalized qRT-PCR values was built with the Pearson coefficient metric and complete linkage. These data allowed good categorization of 16 out of 19 responders (*R*) and 10 out of 13 non-responders (*NR*), for a total of 26 among the 32 patients with RA overall in subset 2. Intensity values were expressed as an expression ratio (*transcript*/housekeeping (*HK*) gene)

We wanted to confirm that a combination of the aforementioned transcript levels was associated with response to ABA/MTX. For this purpose, we used linear discriminant analysis to select a minimal combination of transcripts from “identification” subset 1 that were able to correctly classify patients with RA from “validation” subset 2. Among these 87 mRNA, four transcripts (*BLOC1S1*, *RNASE3*, *COX6A1*, *PTRH2*) were retained by linear discriminant analysis although they did not have the smallest *p* value (Table 2). As shown in Fig. 4, hierarchical clustering of the 32 patients from subset 2, based on the levels of these four transcripts measured by qRT-PCR, resulted in two major clusters of R versus NR, with 26 well-categorized patients. This procedure identified six misclassified patients and indicated that this set of four transcripts provides 75% sensitivity, 85% specificity, and 85% negative predictive value and 75% positive predictive value for identification of future R and NR to ABA/MTX. To ascertain if this signature was specific to ABA, we used this combination of 87 mRNA associated with drug response in an independent cohort of patients with RA, with the same level of disease activity, treated by TNFα blocking agents given as etanercept or adalimumab (Additional file 3), both associated with MTX. This combination was unable to correctly classify R and NR to TNFα blocking agents whatever the molecular structure (fusion protein or monoclonal antibody directed against TNFα), suggesting specificity of this signature for ABA/MTX (Additional file 3). This combination of 87 transcripts also raised the question of understanding the biological significance of these genes in response to ABA/MTX in the pathophysiology of RA.

**Table 2:** Gene ontology (GO) analysis with the 87 transcripts dysregulated between responders and non-responders to abatacept/methotrexate

GO accession	GO term	*P* value	Count in 87 mRNA list	Percentage in 87 mRNA list	Count in human genome	Percentage in human genome	Enrichment
Biological process							
GO:1902600	Hydrogen ion transmembrane transport	0.0011	6	10.17	93	0.55	18.38
GO:0022904	Respiratory electron transport chain	0.0011	6	10.17	99	0.59	17.26
GO:0022900	Electron transport chain	0.0011	6	10.17	101	0.60	16.92
GO:0045333	Cellular respiration	0.0011	7	11.86	146	0.87	13.66
GO:0015992	Proton transport	0.0019	6	10.17	115	0.68	14.86
GO:0006818	Hydrogen transport	0.0019	6	10.17	117	0.70	14.61
Molecular function							
GO:0015002	Heme-copper terminal oxidase activity	0.0011	4	6.78	23	0.14	49.54
GO:0004129	Cytochrome-c oxidase activity	0.0011	4	6.78	23	0.14	49.54
GO:0016676	Oxidoreductase activity, acting on a heme group of donors, oxygen as acceptor	0.0011	4	6.78	23	0.14	49.54
GO:0016675	Oxidoreductase activity, acting on a heme group of donors	0.0011	4	6.78	24	0.14	47.47
GO:0015078	Hydrogen ion transmembrane transporter activity	0.0011	6	10.17	88	0.52	19.42
Cellular localization							
GO:0070469	Respiratory chain	0.0011	6	10.17	68	0.40	25.13
GO:0044455	Mitochondrial membrane part	0.0011	7	11.86	144	0.86	13.85
GO:0005743	Mitochondrial inner membrane	0.0011	10	16.95	350	2.08	8.14
GO:0019866	Organelle inner membrane	0.0011	10	16.95	390	2.32	7.30
GO:0005746	Mitochondrial respiratory chain	0.0017	5	8.47	62	0.37	22.97
GO:0005740	Mitochondrial envelope	0.0017	11	18.64	544	3.24	5.76
GO:0031966	Mitochondrial membrane	0.0053	10	16.95	508	3.02	5.61

Corrected *p* values were determined by a standard hypergeometric distribution

### Pre-silencing of electron transport chain pathway associated with response to ABA/MTX

Different approaches were used to understand the involvement of this 87 mRNA signature in biological processes. These 87 mRNA were submitted to GO analysis leading us to discover enrichment (from 6 to 49 times more) of 18 GO classes in these 87 mRNA compared to the whole human genome (Table 2). Among these 87 transcripts, 11 were associated with these 18 GO classes (Additional file 4).

All these 18 GO classes, based on the 87 mRNA, were relative to the mitochondrial respiratory chain located in the inner membrane of mitochondria (corrected *p* value <0.005). For instance, 6/87 mRNA of genes linked to the electron transport chain (ETC; GO: 0022900) were included in our signature while 101 genes from the whole human genome are known to be relative to this same GO class (corrected *p* value = 0.001). The fold enrichment in this GO term is × 16 (Table 2). Moreover, these 87 mRNA were submitted to WikiPathways using the SEA tool from GeneSpring software leading us to identify enrichment of four signaling pathways: Electron Transport Chain WP111_41171 (version of 1 March 2011; *p* value = 1.6.10-9), Oxidative Phosphorylation WP623_45305 (version of 7 October 2011; *p* value = 2.5.10-4), Proteasome Degradation WP183_45274 (version of 7 October 2011; *p* value = 0.008) and TSH signaling pathway WP2032_44635 (version of 22 September 2011 ; *p* value = 0.008). GO analysis and SEA were in agreement as the ETC pathway localized in mitochondria was found by using these two tools.

**Fig. 5:**
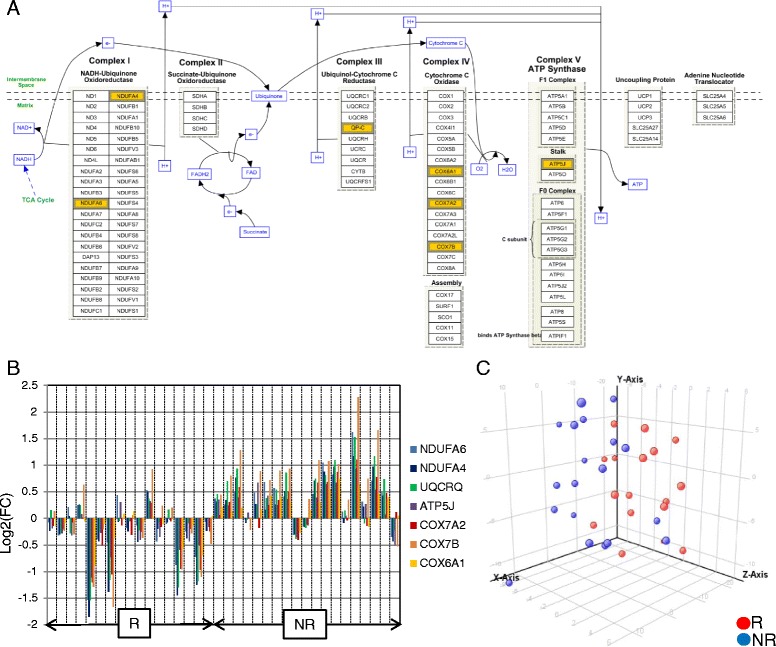
Pre-silencing of mitochondrial respiratory chain associated with abatacept/methotrexate (ABA/MTX) response. **a** Electron transport chain (ETC) WP111-41171 WikiPathway (http://www.wikipathways.org/index.php/Pathway:WP111). In the 87 mRNA dysregulated between responders (*R*) and non-responders (*NR*) to ABA/MTX, there was highly significant enrichment of mRNA involved in ETC (*p* value = 1.6.10^-9^). The *p* value was determined by standard hypergeometric distribution. This enrichment involved 7 mRNA from the 87 transcripts, which are highlighted in *yellow* among the 104 proteins from the whole ETC pathway. **b** Gene expression level expressed as fold change (*FC*) (patients with RA/control pool) for these 7 mRNA in 17 R and 19 NR. **c** Principal component analysis of 17 R and 19 NR performed with gene expression level (fold change) of the 104 mRNA from the whole ETC pathway. Component 1: 40.3%; component 2: 16.7%

As shown in Fig. 5a, the ETC pathway includes 104 proteins split up into five complexes of ETC, embedded in the inner membrane of mitochondria. Among the 87 mRNA of our signature, 7 transcripts from the ETC pathway (*NDUFA6*, *NDUFA4*, *UQCRQ*, *ATP5J*, *COX7A2*, *COX7B*, *COX6A1*) covering four out of five complexes of this pathway, were significantly (*p* value <0.05) downregulated in 17 R versus 19 NR to ABA/MTX (Fig. 5a and b). This differential gene expression profile including these seven genes suggested reduced expression of the mitochondrial respiratory chain pathway in R before ABA administration (Fig. 5b). In addition, taking into account gene expression profiling from the whole ETC pathway (*n* = 104 genes), principal component analysis correctly separated R from NR before treatment, even if all the transcripts from this pathway were not significantly dysregulated between R and NR (Fig. 5c). Nevertheless, this ETC combination including these 104 transcripts did not better separate R and NR (73% specificity) than did the minimal combination of four transcripts (85% specificity) (Fig. 4).

The dysregulation of these transcripts could be linked to the more elevated CRP rate observed in NR compared to R at baseline (Table 1). Correlations between each significant transcript level from the ETC pathway (*NDUFA6*, *NDUFA4*, *UQCRQ*, *ATP5J*, *COX7A2*, *COX7B*, *COX6A1*) and CRP or DAS28(CRP) were performed to discard a CRP or disease activity effect on the gene expression levels from this signature linked to a supposedly more pronounced mitochondrial activity in the inflammatory state. A weak positive coefficient of correlation (*r*) and coefficient of determination (*r* ^2^) between CRP (0.25 < *r* < 0.49; 0.06 < *r* ^2^ < 0.24) or DAS28(CRP) (0.32 < *r* < 0.43; 0.10 < *r* ^2^ < 0.18) and gene expression level was observed for the majority of genes except *ATP5J*. In subset 2, weak positive correlation between CRP or DAS28(CRP) and gene expression data was also found for *NDUFA4*, *COX6A1*, *COX7A2* and *COX7B* (data not shown). All these data led us to discard the impact of CRP or disease activity on the dysregulation of the ETC pathway between R and NR at baseline (Additional file 5).

Finally, we measured gene expression fluctuation in an independent transcriptomic analysis between baseline and 6 months of treatment by ABA/MTX in R and NR. Only five genes (*COX7B*, *COX11*, *UQCRC2*, *NDUFU3*, *NDUFS1*) among the 104 genes involved in the ETC pathway were significantly upregulated in R after 6 months of treatment, while these genes were invariant in NR to ABA/MTX (data not shown). This observation suggests that ABA increased gene expression levels of some genes from the ETC pathway only in R and not in NR independently of disease activity, which decreased whatever the response to ABA/MTX. Overall, these results suggest that these gene levels were not related to disease but rather they are genes of susceptibility to ABA and that silent expression of the ETC signature is associated more with good response to ABA/MTX.

## Discussion

In this ancillary study, we identified 87 transcripts with relative abundances, which were able to separate R and NR to ABA/MTX in 36 patients with RA before treatment (Fig. 3 and Additional file 2). Next, for the first time we validated a minimal combination of four transcripts associated with response to ABA/MTX in an independent subset of 32 patients with RA (Fig. 4). According to GO and WikiPathways analysis, we identified enrichment of 7 genes among the aforementioned 87 belonging to the ETC pathway, suggesting a specific signature of response (Fig. 5). Indeed, patients with RA with pre-silencing of the ETC pathway before treatment were those most susceptible to responding to ABA/MTX 6 months later.

In the current context of personalized medicine for the management of RA, the optimization of drug prescription is crucial. Hence, drug prescription should be tailored exclusively only to patients susceptible to responding to the drug. The identification of biomarkers to predict of drug response is paramount. To date, there are only two biomarkers associated with good response to ABA/MTX in RA, anti-CCP positivity and low baseline number of CD8^+^CD28^–^ T cells [27–30]. Furthermore, there is no clinical and/or biological biomarker used in routine practice able to predict response to ABA/MTX prior to treatment initiation. Indeed, in our study, two statistical approaches to multivariate analysis were unable to identify predictive clinical or biological parameters of response to ABA/MTX, even if disease duration, CRP, global patient assessment of disease and DAS28(CRP) were significantly different between R and NR at baseline (Table 1 and Fig. 2). It would be interesting to establish a relationship between response to ABA and anti-citrullinated protein antibodies (ACPA) and/or rheumatoid factor status, given that ACPA positivity is associated with better response to ABA in RA. Unfortunately, we were unable to include ACPA or rheumatoid factor status in our analysis because these data were not collected during the APPRAISE trial and were not available. The difficulties we faced in predicting drug response with clinical and/or biological variables led us to use a more global functional genomic approach without a priori having to identify a signature associated with good response to ABA/MTX.

The transcriptomic approach based on the whole genome allowed us to identify signatures able to separate R from NR to several drugs (infliximab, tocilizumab, rituximab) used in RA [17–21, 31]. For ABA, one study previously measured the type-I interferon-regulated transcripts from peripheral blood mononuclear cells in patients with RA, independently of response to ABA [32]. Another study identified gene sets associated with remission according to clinical disease activity index (CDAI) in ABA-treated patients with RA [33]. In our study, even if there were differences at baseline in disease duration, CRP, patient assessment of disease and DAS28(CRP) between R and NR, which might have influenced gene expression, we identified and validated a gene signature associated with response to ABA/MTX. This signature included 87 genes variously involved in the ETC, proteasome, interferon and RNA processes, etc. This combination of 87 genes is also specific to ABA as it was not able to predict TNFα blocking agent response. Next, we validated this signature with an independent subset of 32 patients with RA by means of a minimal combination of four genes with expression levels measured by qRT-PCR, which are more easily usable in routine practice than microarrays. This signature is the optimized combination of genes associated with drug response with good accuracy, because it correctly predicted the future response in 81% of patients with RA (75% sensitivity, 85% specificity, 85% negative predictive value and 75% positive predictive value) prior to treatment (Fig. 4). Each gene taken separately was unable to predict response to ABA/MTX (data not shown) but all genes together were associated with response to ABA/MTX with good accuracy. Nevertheless, further validation studies in independent cohorts are essential before considering this signature as a predictive biomarker and of use in clinical practice.

Of the four genes in the minimal combination, the *RNAseIII* gene codes for the RNAseIII enzyme that specifically cleaves double-stranded RNA and is involved in the processing of ribosomal RNA precursors of some mRNAs [34]. Biogenesis of lysosomal organelles complex-1, subunit 1 (*BLOC1S1*) codes for the protein BLOC1S1, also known as GCN5L1, and is an essential component of the mitochondrial acetyltransferase machinery and modulates mitochondrial respiration via acetylation of ETC proteins [35]. *COX6A1* codes for the mitochondrial protein cytochrome c oxidase subunit 6A1 (COX6A1) located in complex IV. This is the last enzyme in the mitochondrial ETC that drives ATP synthesis [36]. *PTRH2* codes for the peptidyl-tRNA hydrolase 2, which is a mitochondrial protein released from mitochondria to the cytoplasm during apoptosis.

Out of these 4 transcripts, 3 (*BLOC1S1*, *COX6A1* and *PTRH2*) and 13 probes out of 87 code for proteins located in mitochondria. In addition, some of them are involved in the ETC pathway, suggesting implication of mitochondrial metabolism in response to ABA/MTX. Moreover, GO analysis and SEA are in agreement as these two analyses point to the involvement of ETC in the response to ABA/MTX. Interestingly, we found that seven transcripts with levels that are significantly lower in R than in NR at baseline were similarly regulated in patients in remission and patients not in remission defined by clinical disease activity index (CDAI) in a recent study [33]. The ETC is a series of five complexes anchored to the inner membrane of mitochondria that transfers electrons via redox reactions, which drives ATP synthesis, generating reactive oxygen species (ROS) and subsequent oxidative stress [37]. Redox balance in mitochondria is a critical component in T cell activation and proliferation [38]. The production of ROS by the ETC complex III leads to production of large amounts of ATP to enhance activity of proliferating T cells after TCR cross-linking [39–42]. In our study, ETC genes were downregulated in R compared to NR, suggesting potentially low ROS production and potentially less T cell activation before treatment. Also, a previous study suggested it would be interesting to determine whether ROS production in T cells might be a predictor of clinical response to ABA [43]. Our results suggest the possible involvement of ROS in the pathophysiological processes of ABA response, but further functional studies are necessary to confirm this hypothesis.

This 87 mRNA signature also included *RASSF5*, which was significantly upregulated in R compared to NR. *RASSF5*, also known as *RAPL,* is the effector of Rap1, which plays a central role in T cell response through TCR and co-stimulation signals. Indeed, a model was proposed in which inactivation of Rap1 plays a central role in establishing oxidative stress and can influence T cell response in RA [43, 44]. These data suggest less oxidative stress in future responders to ABA while NR present with high expression of genes from the ETC pathway, showing oxidative stress. A reduction in the expression level of ETC genes seems to increase the sensitivity of patients with RA to ABA/MTX. This model has already been highlighted in esophageal adenocarcinoma and colorectal cancer treated by chemotherapy [45, 46]. Indeed, *ATP5J* and *COX7A2* included in our combination were also found to be downregulated and associated with response to chemotherapy, respectively in colorectal cancer and esophageal adenocarcinoma [45, 46]. As in cancer, pre-treatment conditions targeting the mitochondrial metabolism might be a determinant of susceptibility to ABA/MTX [47].

After 6 months of treatment with ABA, we showed that five genes (*COX7B*, *COX11*, *UQCRC2*, *NDUFU3*, *NDUFS1*) involved in the ETC pathway were significantly upregulated in R, while these genes were invariant in NR to ABA/MTX. So, whereas ABA does not seem to affect the oxidative state in NR, it seems to modulate oxidative stress in R, as genes from the ETC pathway were upregulated under ABA. This drug restores the expression of genes involved in redox balance.

## Conclusions

In summary, we identified and validated a gene expression signature associated with good response to ABA/MTX. This signature involves genes from the ETC pathway. The pre-silencing of these genes could be a specific determinant of susceptibility to MTX + ABA. The downregulation of these genes reflected the redox imbalance observed in R. The intracellular redox balance is crucial for the antigenic response of T cells. After 6 months of treatment, ABA significantly upregulated some ETC genes in R probably leading to a slight increase in ROS, restoring the redox balance and improving T cell response. Further studies, including the investigation of ROS levels in R and NR to ABA/MTX, are necessary for large-scale validation of this signature before evaluating its pertinence in clinical practice.

## Additional files

Additional file 1: Variation in clinical and biological parameters between baseline and 6 months according to response (PDF 273 kb)

Additional file 2: Transcripts differentially expressed in ABA/MTX responders (*R*) vs non-responders (*NR*) (PDF 305 kb)

Additional file 3: Ineffectiveness of 87 mRNA associated with response to ABA/MTX to predict response to adalimumab or etanercept (PDF 49 kb)

Additional file 4: Transcripts associated with Gene Ontology enrichment analysis (PDF 225 kb)

Additional file 5: Weak correlation between inflammatory state or disease activity and gene expression level from the ETC pathway at baseline (PDF 201 kb)

## Abbreviations

ABA: Abatacept
ACPA: Anti-citrullinated protein antibodies
ACR: American College of Rheumatology
ATP: Adenosine triphosphate
ATP5J: ATP Synthase H+ transporting Mitochondrial Fo Complex
BLOC1S1: Biogenesis of lysosomal organelles complex-1 subunit 1
CDAI: Clinical Disease Activity Index
COX11: Cytochrome C oxidase subunit 1
COX6A1: Cytochrome c oxidase subunit 6A1
COX7A2: Cytochrome C oxidase subunit VIIa polypeptide 2
COX7B: Cytochrome C oxidase subunit VIIb
cRNA: Complementary ribonucleic acid
CRP: C-reactive protein
DAS: Disease Activity Score
ETC: Electron transport chain
EULAR: European League Against Rheumatism
FDR: False discovery rate
GCN5L1: General control of amino acid synthesis 5 like 1
GO: Gene Ontology
IL1-Ra: Interleukin 1 receptor antagonist
mAb: Monoclonal antibody
mRNA: Messenger ribonucleic acid
MTX: Methotrexate
NDUFA4: NADH Dehydrogenase ubiquinone 1 alpha subcomplex 4
NDUFA6: NADH dehydrogenase ubiquinone 1 alpha subcomplex 6
NDUFS1: NADH ubiquinone oxidoreductase core subunit S1
NDUFU3: NADH dehydrogenase-ubiquinone Fe-S subunit 3
NR: Non-responder
PDUS: Power Doppler grayscale ultrasound
PSMA6: Proteasome subunit alpha type 6
PSMB6: Proteasome subunit beta type-6
PTRH2: Peptidyl-tRNA hydrolase 2
qRT-PCR: Quantitative reverse-transcription polymerase chain reaction
R: Responder
RA: Rheumatoid arthritis
Rap1: Ras-related protein 1
RASSF5: Ras association domain family member 5
RNASE3: Ribonuclease 3
ROS: Reactive oxygen species
SEA: Single Experiment Analysis
TJC: tender joint count
TNF-α: Tumor necrosis factor alpha
UBL5: Ubiquitin like 5
UQCRC2: Ubiquinol cytochrome C reductase complex core protein 2
UQCRQ: Ubiquinol-cytochrome C reductase complex III subunit VII
VAS: Visual analog scale
