# Supplementary material for: Pre-silencing of genes involved in the electron transport chain (ETC) pathway is associated with responsiveness to abatacept in rheumatoid arthritis
Source: Arthritis Res Ther. 2017 May 25;19:109. doi: 10.1186/s13075-017-1319-8 (PMC5445375; doi:10.1186/s13075-017-1319-8)
Supplement: Supplementary file 1 — Variation in clinical and biological parameters between baseline and 6 months according to response (PDF 273 kb) [file 13075_2017_1319_MOESM1_ESM.pdf]

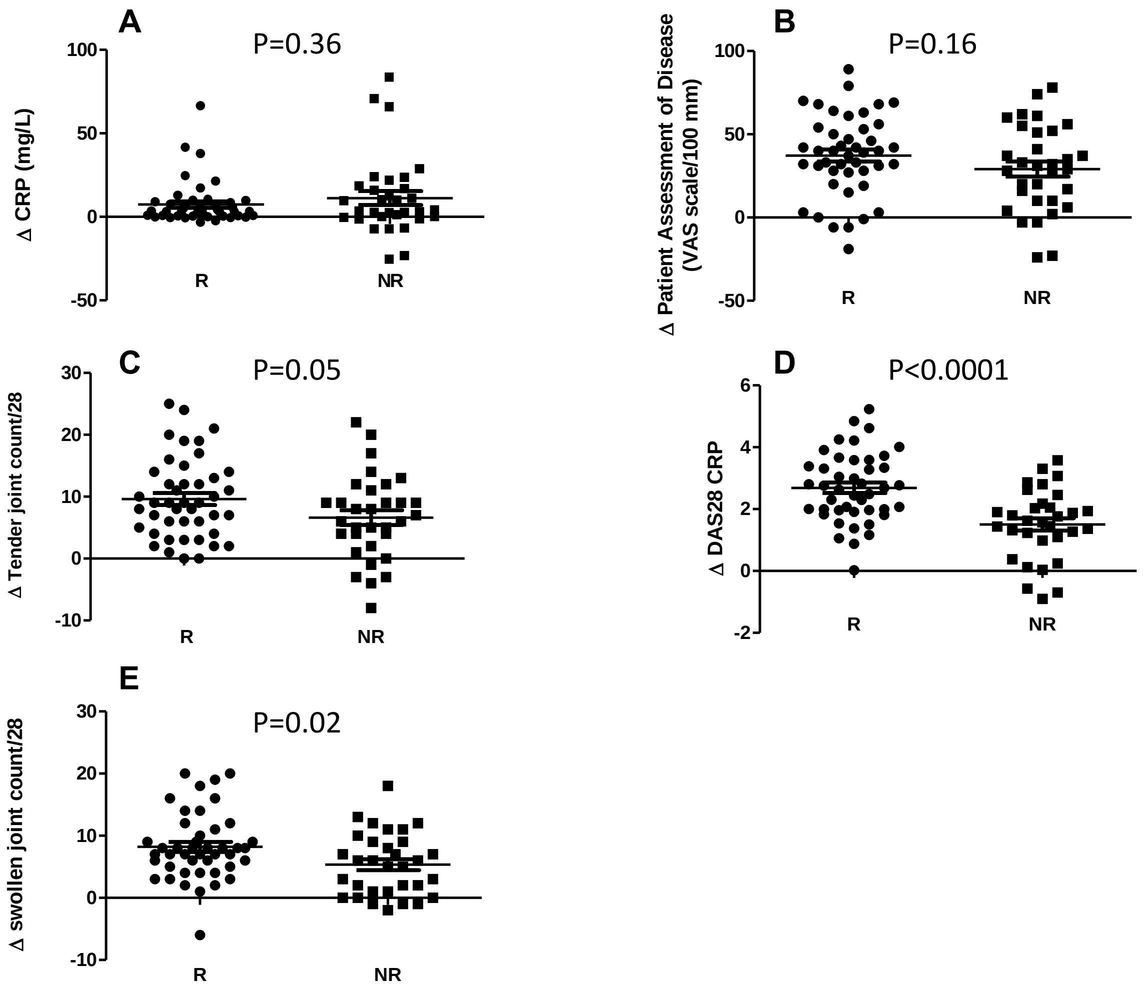

<u>Additional file 1:</u> Variation of clinical and biological parameters between baseline and 6 months according to response. Variations between baseline and 6 months were plotted for each parameters: C reactive protein (CRP) (**A**), patient assessment of disease (**B**), number of tender joint count/28 (**C**), DAS28(CRP) (**D**), and number of swollen joint count/28 (**E**). n=68 RA patients (36 R and 32 NR) and *p.* Values were obtained by t-test. DAS28: disease activity score. VAS: visual analog scale.
