# Supplementary material for: Pre-silencing of genes involved in the electron transport chain (ETC) pathway is associated with responsiveness to abatacept in rheumatoid arthritis
Source: Arthritis Res Ther. 2017 May 25;19:109. doi: 10.1186/s13075-017-1319-8 (PMC5445375; doi:10.1186/s13075-017-1319-8)
Supplement: Supplementary file 2 — Transcripts differentially expressed in ABA/MTX responders (R) vs non-responders (NR) (PDF 305 kb) [file 13075_2017_1319_MOESM2_ESM.pdf]

Agilent probe ID	P (Corr)	FC	Gene Symbol	GeneName	RefSeqAcc
A_23_P163025	0.00001	2.36	RNASE3	ribonuclease, RNase A family, 3	NM_002935
A_32_P1712	0.00001	2.39	RNASE2	ribonuclease, RNase A family, 2 (liver, eosinophil-derived neurotoxin)	NM_002934
A_23_P151637	0.00003	2.44	RNASE2	ribonuclease, RNase A family, 2 (liver, eosinophil-derived neurotoxin)	NM_002934
A_24_P261929	0.019	1.43	IFI27L1	interferon, alpha-inducible protein 27-like 1	NM_206949
A_23_P78134	0.022	1.24	EMC6	ER membrane protein complex subunit 6	NM_001014764
A_23_P328511	0.022	1.40	HSBP1	heat shock factor binding protein 1	NM_001537
A_23_P78563	0.022	1.43	UBL5	ubiquitin-like 5	NM_024292
A_23_P53298	0.022	1.48	BLOC1S1	biogenesis of lysosomal organelles complex-1, subunit 1	NM_001487
A_23_P417632	0.022	1.94	ECRP	ribonuclease, RNase A family, 2 pseudogene	NR_033909
A_23_P75769	0.025	2.17	MS4A4A	membrane-spanning 4-domains, subfamily A, member 4A	NM_024021
A_23_P130642	0.027	-1.24	GBP6	guanylate binding protein family, member 6	NM_198460
A_24_P88505	0.027	1.44	ZCCHC17	zinc finger, CCHC domain containing 17	NM_016505
A_23_P20384	0.027	1.45	LSM1	LSM1, U6 small nuclear RNA associated	NM_014462
A_23_P76488	0.028	1.93	EMP1	epithelial membrane protein 1	NM_001423
A_23_P358555	0.030	1.31	NAA38	N(alpha)-acetyltransferase 38, NatC auxiliary subunit	NM_032356
A_23_P209347	0.030	-1.36	ANKRD44	ankyrin repeat domain 44	NM_153697
A_23_P25735	0.030	1.56	PSMA6	proteasome (prosome, macropain) subunit, alpha type, 6	NM_002791
A_23_P81690	0.030	1.59	COX7A2	cytochrome c oxidase subunit VIIa polypeptide 2 (liver)	NM_001865
A_32_P98313	0.030	1.79	NDUFA4	NADH dehydrogenase (ubiquinone) 1 alpha subcomplex, 4, 9kDa	NM_002489
A_23_P52647	0.031	-1.27	EHD1	EH-domain containing 1	NM_006795
A_23_P154832	0.031	1.47	ATP5J	ATP synthase, H+ transporting, mitochondrial Fo complex, subunit F6	NM_001003703
A_23_P425932	0.032	-1.27	VTI1A	vesicle transport through interaction with t-SNAREs 1A	XM_005269544
A_32_P168247	0.032	1.34	COX6A1	cytochrome c oxidase subunit VIa polypeptide 1	NM_004373
A_23_P15493	0.032	1.21	PTRH2	peptidyl-tRNA hydrolase 2	NM_016077
A_23_P98382	0.032	1.66	TIMM8B	translocase of inner mitochondrial membrane 8 homolog B (yeast)	NM_012459
A_23_P74114	0.034	-1.22	ZNF713	zinc finger protein 713	NM_182633
A_23_P140146	0.034	1.47	IFI27L2	interferon, alpha-inducible protein 27-like 2	NM_032036
A_23_P159650	0.035	2.01	COX7B	cytochrome c oxidase subunit VIIb	NM_001866
A_23_P1254	0.036	1.35	GSTO1	glutathione S-transferase omega 1	NM_004832

A_24_P113815	0.036	-1.36	SLC35E2	solute carrier family 35, member E2	NM_182838
A_23_P132644	0.036	1.40	NCEH1	neutral cholesterol ester hydrolase 1	NM_020792
A_23_P41114	0.036	2.08	CSTA	cystatin A (stefin A)	NM_005213
A_32_P131377	0.037	1.26	REEP5	receptor accessory protein 5	NM_005669
A_23_P127533	0.037	1.39	DCUN1D5	DCN1, defective in cullin neddylation 1, domain containing 5	NM_032299
A_24_P186379	0.037	1.34	FUOM	fucose mutarotase	NM_198472
A_23_P145777	0.038	1.76	NDUFA4	NADH dehydrogenase (ubiquinone) 1 alpha subcomplex, 4, 9kDa	NM_002489
A_32_P43217	0.038	1.52	PSMA6	proteasome (prosome, macropain) subunit, alpha type, 6	NM_002791
A_23_P213718	0.038	1.77	UQCRQ	ubiquinol-cytochrome c reductase, complexIII	NM_014402
A_24_P304051	0.039	1.35	GSTO1	glutathione S-transferase omega 1	NM_004832
A_23_P170233	0.039	1.91	CSTA	cystatin A (stefin A)	NM_005213
A_23_P59877	0.039	1.49	FABP5	fatty acid binding protein 5 (psoriasis-associated)	NM_001444
A_23_P59022	0.040	-1.20	TRERF1	transcriptional regulating factor 1	NM_033502
A_23_P201497	0.040	-1.43	RASSF5	Ras association (RalGDS/AF-6) domain family member 5	NM_182663
A_23_P128734	0.040	1.47	ERH	enhancer of rudimentary homolog (Drosophila)	NM_004450
A_32_P205553	0.040	1.51	RPL26L1	ribosomal protein L26-like 1	NM_016093
A_23_P380848	0.040	1.54	TXNDC17	thioredoxin domain containing 17	NM_032731
A_23_P344973	0.040	1.55	MYL6	myosin, light chain 6, alkali, smooth muscle and non-muscle	NM_079423
A_23_P326080	0.040	4.47	DEFA4	defensin, alpha 4, corticostatin	NM_001925
A_23_P26413	0.040	-1.32	RBL2	retinoblastoma-like 2	NM_005611
A_32_P187827	0.040	-1.38	NPIPB9	nuclear pore complex interacting protein family, member B9	NM_001287251
A_23_P79199	0.040	1.64	DBI	diazepam binding inhibitor	NM_020548
A_23_P114929	0.041	1.18	MPC2	mitochondrial pyruvate carrier 2	NM_015415
A_23_P127467	0.041	1.22	EIF1AD	eukaryotic translation initiation factor 1A domain containing	NM_032325
A_23_P114282	0.041	1.47	MCTS1	malignant T cell amplified sequence 1	NM_014060
A_23_P353014	0.041	1.37	CACNA2D4	calcium channel, voltage-dependent, alpha 2/delta subunit 4	NM_172364
A_23_P371613	0.041	1.32	CHCHD1	coiled-coil-helix-coiled-coil-helix domain containing 1	NM_203298
A_23_P380240	0.041	3.85	CEACAM8	carcinoembryonic antigen-related cell adhesion molecule 8	NM_001816
A_23_P42375	0.043	1.26	RAB32	RAB32, member RAS oncogene family	NM_006834
A_23_P15705	0.043	1.32	PSMB6	proteasome (prosome, macropain) subunit, beta type, 6	NM_002798

A_23_P71148	0.044	1.42	BLVRA	biliverdin reductase A	NM_000712
A_24_P23245	0.044	1.57	NDUFA6	NADH dehydrogenase (ubiquinone) 1 alpha subcomplex, 6, 14kDa	NM_002490
A_23_P54179	0.045	1.15	ZNF410	zinc finger protein 410	NM_021188
A_23_P103282	0.048	1.22	TMEM59	transmembrane protein 59	NM_004872
A_23_P7087	0.048	-1.14	CTBP1-AS2	CTBP1 antisense RNA 2 (head to head)	NR_033339
A_23_P326170	0.048	1.28	CALM2	calmodulin 2 (phosphorylase kinase, delta)	NM_001743
A_23_P63178	0.049	1.26	TAF12	TAF12 RNA polymerase II, TATA box binding protein (TBP)-associated factor	NM_005644
A_23_P7697	0.049	1.28	SNX2	sorting nexin 2	NM_003100
A_23_P97005	0.049	-1.42	JAK1	Janus kinase 1	NM_002227
A_23_P205281	0.050	1.39	C14orf2	chromosome 14 open reading frame 2	NM_004894

Additional file 2: **Transcripts differentially expressed in ABA/MTX responders (R)** ***vs*** **non-responders (NR).** Only Agilent's probes with accession number (RefSeqAcc) are reported in this table (69 out of 87 transcripts). Fold change (FC) is the ratio of relative abundance of transcripts in NR *versus* R. Positive FC means an up-regulation of the corresponding transcript in NR patients and conversely. *P* values were determined by t-test with Benjamini-Hochberg correction for false discovery rate estimation ($p < 0.05$).
