# Supplementary material for: Pre-silencing of genes involved in the electron transport chain (ETC) pathway is associated with responsiveness to abatacept in rheumatoid arthritis
Source: Arthritis Res Ther. 2017 May 25;19:109. doi: 10.1186/s13075-017-1319-8 (PMC5445375; doi:10.1186/s13075-017-1319-8)
Supplement: Supplementary file 3 — Ineffectiveness of 87 mRNA associated with response to ABA/MTX to predict response to adalimumab or etanercept (PDF 49 kb) [file 13075_2017_1319_MOESM3_ESM.pdf]

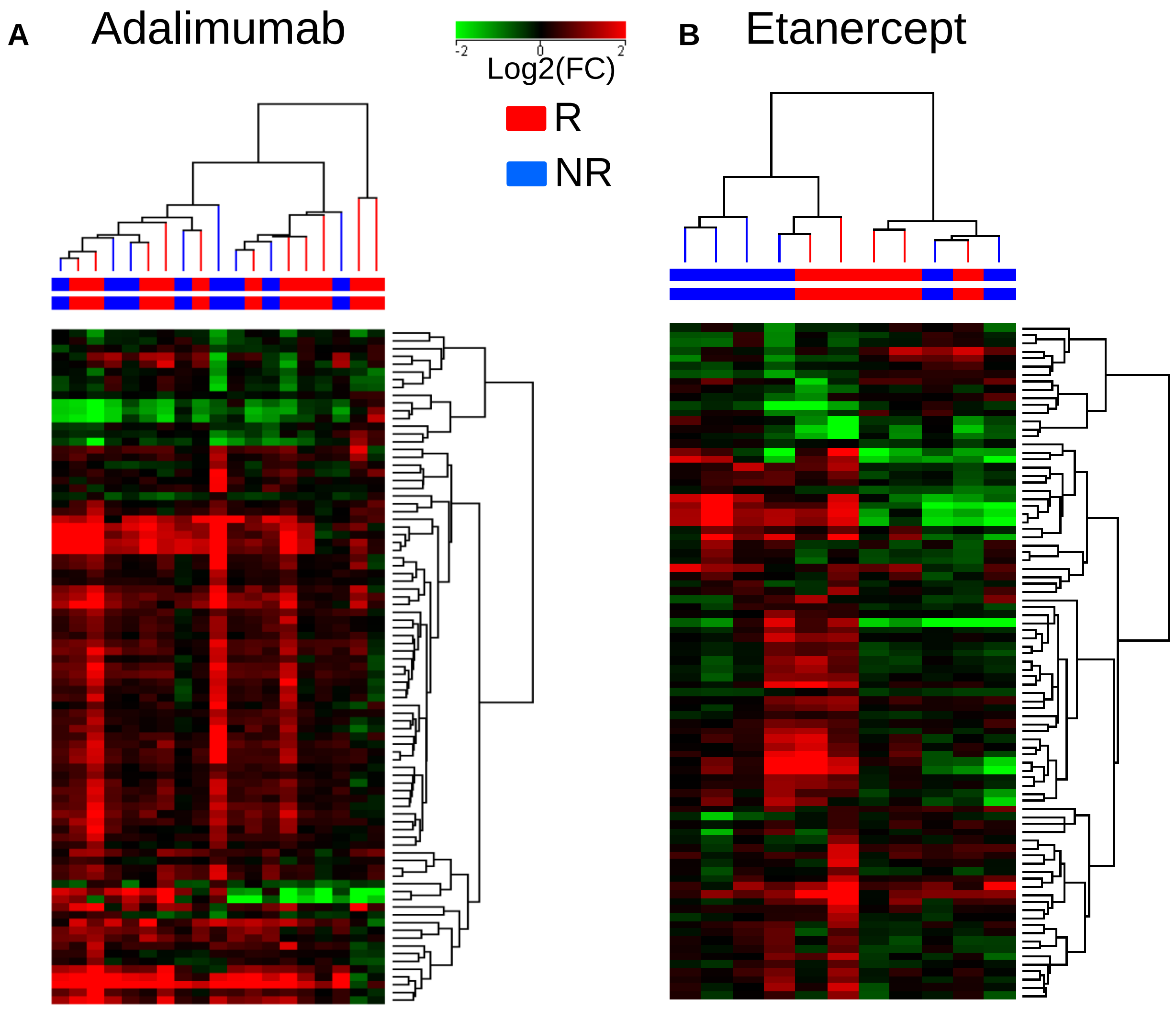

<u>Additional file 3: </u>Ineffectiveness of 87mRNA associated with response to ABA/MTX to predict response to adalimumab or etanercept. Hierarchical clustering performed with 87 mRNA associated to MTX/abatacept response were performed with two independent cohorts of RA patients treated by adalimumab or etanercept. (**A**) Nineteen RA patients were enrolled in adalimumab study including 11 responders (R) and 8 non-responders (NR). (**B**) Eleven RA patients were enrolled in etanercept study including 5 R and 6 NR. Pearson coefficient metric was used to obtain the dendrograms. Adalimumab or etanercept transcriptomic data were obtained in the same conditions and processed as abatacept transcriptomic data (4x44K whole human genome microarrays from Agilent). Values were expressed as a fold change (RA patient/healthy control).
