# Supplementary material for: Pre-silencing of genes involved in the electron transport chain (ETC) pathway is associated with responsiveness to abatacept in rheumatoid arthritis
Source: Arthritis Res Ther. 2017 May 25;19:109. doi: 10.1186/s13075-017-1319-8 (PMC5445375; doi:10.1186/s13075-017-1319-8)
Supplement: Supplementary file 4 — Transcripts associated with Gene Ontology enrichment analysis (PDF 225 kb) [file 13075_2017_1319_MOESM4_ESM.pdf]

GeneSymbol	GeneName	RefSeq Accession	Go class number	Evidence Code (contributors)
ATP5J	ATP synthase, H+ transporting, mitochondrial Fo complex, subunit F6	NM_0010037 03	GO:0022900	IDA (UniProt) TAS (reactome)
BLOC1S1	biogenesis of lysosomal organelles complex-1, subunit 1	NM_001487	GO:0045333	IMP (UniProt)
C14orf2	chromosome 14 open reading frame 2	NM_004894	GO:0031966	ISS (UniProt)
COX6A1	cytochrome c oxidase subunit VIa polypeptide 1	NM_004373	GO:0022900	TAS (reactome)
COX7A2	cytochrome c oxidase subunit VIIa polypeptide 2	NM_001865	GO:0031966	TAS (reactome) IEA (InterPro)
COX7B	cytochrome c oxidase subunit VIIb	NM_001866	GO:0022900	TAS (reactome)
MPC2	mitochondrial pyruvate carrier 2	NM_015415	GO:0031966	IBA (GO_Central)
NDUFA4	NDUFA4, mitochondrial complex associated	NM_002489	GO:0031966	IDA (UniProt) TAS (reactome)
NDUFA6	NADH dehydrogenase (ubiquinone) 1 alpha subcomplex, 6, 14kDa	NM_002490	GO:0031966	IDA (UniProt) TAS (reactome)
TIMM8B	translocase of inner mitochondrial membrane 8 homolog B	NM_012459	GO:0031966	IEA (InterPro)
UQCRQ	ubiquinol-cytochrome c reductase, complex III subunit VII, 9.5kDa	NM_014402	GO:0022900	TAS (reactome)

Additional file 4: Transcripts associated with GeneOntology enrichment analysis. Eleven transcripts out of 87 significantly dys-regulated between R and NR at baseline were associated with 18 GeneOntology classes significantly enriched. Gene symbol, gene full name, RefSeq accession number, GO class number and evidence code (according to the contributors [UniProt, Reactome, GO_Central, InterPro] which links gene and GO class are indicated. IBA : Inferred from Biological aspect of Ancestor (Computational source of evidence), IDA : Inferred from Direct Assay (Experimental source of evidence), IEA : Inferred from Electronic Assay (Computational source of evidence), IMP : Inferred from Mutant Phenotype (Experimental source of evidence), ISS : Inferred from sequence or structural similarity (Computational source of evidence), TAS : Traceable Author Statement (Indirectly derived from experimental or computational evidence made by the author of the published article).
