# Supplementary material for: Pre-silencing of genes involved in the electron transport chain (ETC) pathway is associated with responsiveness to abatacept in rheumatoid arthritis
Source: Arthritis Res Ther. 2017 May 25;19:109. doi: 10.1186/s13075-017-1319-8 (PMC5445375; doi:10.1186/s13075-017-1319-8)
Supplement: Supplementary file 5 — Weak correlation between inflammatory state or disease activity and gene expression level from the ETC pathway at baseline (PDF 201 kb) [file 13075_2017_1319_MOESM5_ESM.pdf]

A

	NDUFA4	NDUFA6	UQCRQ	ATP5J	COX6A1	COX7A2	COX7B
**R**	0.36	0.39	0.31	0.49	0.31	0.25	0.37
**R^2**	0.13	0.16	0.10	0.24	0.09	0.06	0.14

B

	NDUFA4	NDUFA6	UQCRQ	ATP5J	COX6A1	COX7A2	COX7B
**R**	0.34	0.39	0.34	0.43	0.36	0.32	0.36
**R^2**	0.11	0.15	0.11	0.18	0.13	0.10	0.13

Additional file 5: Low correlation between inflammatory state or disease activity and gene expression level from the ETC pathway at baseline.

Pearson correlation coefficient (R) and coefficient of determination (R^2) were calculated for each gene expression level (Fold Change) for the 7 mRNA from "electron transport chain" pathway significantly dysregulated between R and NR and CRP rate (mg/mL) (**A**) or DAS28(CRP) (**B**). n=36 rheumatoid arthritis patients (subset 1). R: responder, NR: non-responders, CRP: C reactive protein, DAS28: Disease activity score 28.
